# Recognition of Immune Microenvironment Landscape and Immune-Related Prognostic Genes in Breast Cancer

**DOI:** 10.1155/2020/3909416

**Published:** 2020-11-15

**Authors:** Huiling Wang, Shuo You, Meng Fang, Qian Fang

**Affiliations:** The Fifth Department of General Surgery, Hunan Provincial People's Hospital, Changsha, Hunan, China

## Abstract

**Background:**

Breast cancer (BC) is the most common malignant tumor in women. The immunophenotype of tumor microenvironment (TME) has shown great therapeutic potential in tumor.

**Method:**

The transcriptome was obtained from TCGA and GEO data. Immune infiltration was analyzed by single-sample gene set enrichment (ssGSEA). The immune feature was constructed by Cox regression analysis. In addition, the coexpression of differential expression genes (DEGs) was identified. Through enrichment analysis, the function and pathway of module genes were identified. The somatic mutations related to immune characteristics were analyzed by Maftools. By using the consistency clustering algorithm, the molecular subtypes were constructed, and the overall survival time (OS) was predicted.

**Results:**

Immune landscape can be divided into low immune infiltration and high immune infiltration. Cox regression analysis identified seven immune cells as protective factors of BC. In the coexpression modules for DEGs of high and low immune infiltration, module 1 was related to T cells and high immune infiltration. In particular, the area under the curve (AUC) value of hub gene SASH3 was the highest, and the correlation with T cells was stronger in the high immune infiltration. Enrichment analysis found that oxidative stress, T cell aggregation, and apoptosis were observed in high immune infiltration. In addition, TP53 was identified as the most important somatic gene mutation related to immune characteristics. Importantly, we also constructed seven immune cell-based breast cancer subtypes to predict OS.

**Conclusion:**

We evaluated the immune landscape of BC and constructed the gene characteristics related to the immune landscape. The potential of T cells and SASH3 in immunotherapy of BC was revealed, which may guide the development of new clinical treatment strategies.

## 1. Introduction

Breast cancer is a disease in which breast cells grow out of control and eventually form tumors [1]. Breast cancer is one of the most common cancer types in the world with high mortality and malignancy [2]. In 2012, about 522000 women died from breast cancer worldwide [3]. The survival rate after diagnosis varies greatly among breast cancer patients, even those closely matched with tumor characteristics [4]. At present, it is generally believed that the tumor microenvironment (TME) affects the occurrence and development of tumors [5]. Therefore, it is necessary to extract the prognostic factors from the tumor microenvironment, especially the immune microenvironment.

TME immunophenotype plays an important role in predicting clinical efficacy and therapeutic effect [6]. At present, it is generally believed that the presence of immune microenvironment can inhibit tumor growth and prevent tumor metastasis [7]. The weak immunogenicity and strong immunosuppressive environment of breast cancer limit immunotherapy for adaptive immune system, such as checkpoint inhibitor [8]. The infiltrative immune components of breast tumors have been used as biomarkers for prognosis and prediction of chemotherapy and radiotherapy [9, 10].

In breast cancer, high immune infiltration is associated with better clinical efficacy [11]. Especially the degree and type of T cell infiltration affect the prognosis of breast cancer. Some studies in many human cancers have shown that the presence of T cell infiltration is often related to good prognosis [12, 13]. In addition, high immune infiltration was associated with increased response to neoadjuvant and adjuvant chemotherapy [14].

In this study, we used single-sample gene set enrichment (ssGSEA) to analyze the high and low immune cell infiltration in breast cancer. The differentially expressed genes between high and low immune infiltration were analyzed, and the molecular mechanism of different immune infiltration was revealed.

## 2. Materials and Methods

### 2.1. Data Source and Standardization

We collected the original microarray data of breast cancer tissue from The Cancer Genome Atlas (TCGA) and gene expression omnibus (GEO), as well as the relevant clinicopathological data. Among them, TCGA contains 1027 tumor samples, GSE42568 contains 104 breast cancer samples, GSE37751 contains 61 breast cancer samples, and GSE7390 contains 198 breast cancer samples. All data acquisition and analysis are completed with R (3.2.2). Normalization was performed to correct for sample-related differences using R package of EDASeq and quantile normalized using preprocessCore.

### 2.2. Single-Sample Gene Set Enrichment Analysis (ssGSEA)

The marker gene set for immune cell types was obtained from Bindea et al. [15]. The infiltration levels of immune cells were quantified by ssGSEA using gsva package [16]. Tumors with different patterns of immune cell infiltration were classified by unsupervised clustering.

### 2.3. Differentially Expressed Genes

Differentially expressed genes (DEGs) in high and low immune infiltration were performed by Limma package [17, 18] in GEO datasets and DESeq 2 package [19] in TCGA. Set threshold *P* < 0.05.

### 2.4. Weighted Correlation Network Analysis (WGCNA)

The WGCNA package [20] was used to construct the coexpression network. The soft threshold power of *β* is calculated by scale-free topological criterion; then, a weighted adjacency matrix is generated. In addition, the correlation between these modules and immune cells was studied with the Pearson correlation.

### 2.5. Enrichment Analysis and Gene Set Enrichment Analysis (GSEA)

Enrichment analysis of module genes for Gene Ontology (GO) and The Kyoto Encyclopaedia of Genes and Genomes (KEGG) pathway using clusterProfiler package [21]. *P* < 0.05 was the threshold used for the significant terms.

GSEA analysis was used to detect whether the genes with high and low immune infiltration contained significant KEGG pathway.

### 2.6. Somatic Mutation Analysis

The somatic mutation of breast cancer with different immune infiltration in TCGA was calculated by Maftools, using ggplot2 package [22, 23] to draw the distribution map of mutation.

### 2.7. Immunophenotyping

Consensus clustering based on seven immune cells was carried out using the ConsensusClusterPlus package. After that, we used the survival package to conduct Kaplan Meier survival analysis in each cluster.

## 3. Results

### 3.1. Immune Microenvironment in Breast Cancer

**Figure 1:**
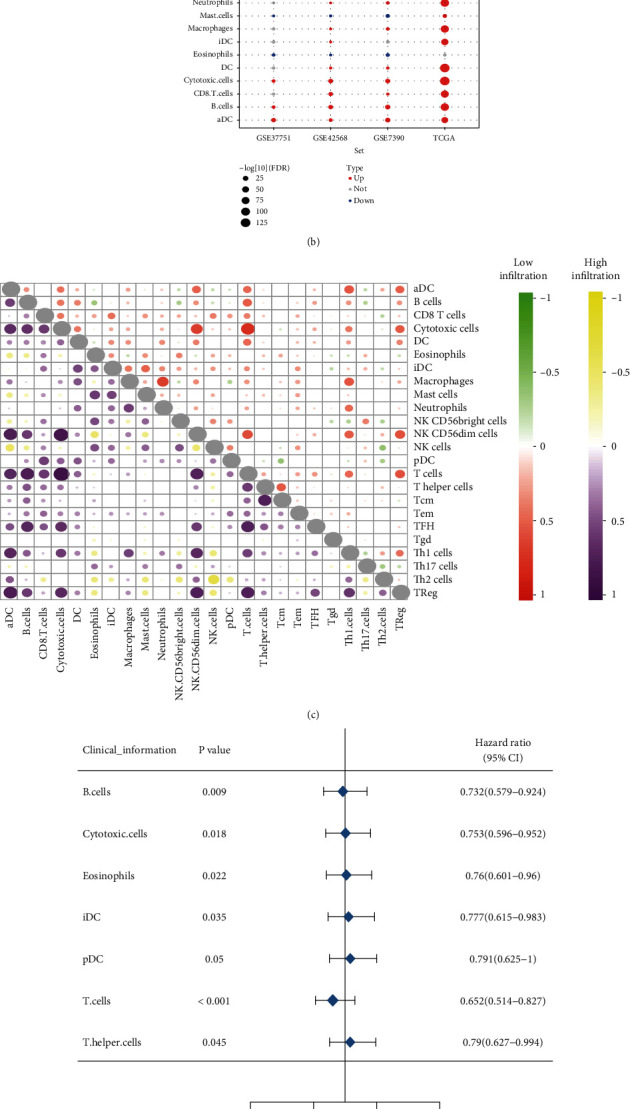
Breast cancer immune cell microenvironment. (a) Classification of high and low immune cell infiltration in breast cancer samples. (b) The difference level of 24 kinds of immune cells in breast cancer with high and low immune cell infiltration. (c) Correlation between immune cells in high or low immune cell infiltration samples. (d) Cox regression analysis of potential prognostic factors of breast cancer. (e) Nomogram of immune cells predicting survival time of breast cancer.

We calculated the infiltration of immune cells of breast cancer samples by ssGSEA into high immune cell infiltration and low immune cell infiltration (Figure 1(a)). There was a significant difference between the high and low immune infiltration groups (Figure 1(b)). In particular, T cells, B cells, DC, and cytotoxic cells had the same direction of difference among the four datasets. The correlation between cytotoxic cells and T cells was the highest in high or low cell infiltration samples (Figure 1(c)). Cox proportional hazards model showed that 7 immune cells were protective factors for survival (B cells, Cytotoxic cells, Eosinophils, iDC, PDC, T cells, and T helper cells) (Figure 1(d)). We constructed a nomogram of immune cells that affect the survival of breast cancer patients, which suggested that T-cell-mediated immune response may prolong the survival time of breast cancer patients (Figure 1(e)).

### 3.2. Differentially Expressed Genes in High and Low Immune Scores

**Figure 2:**
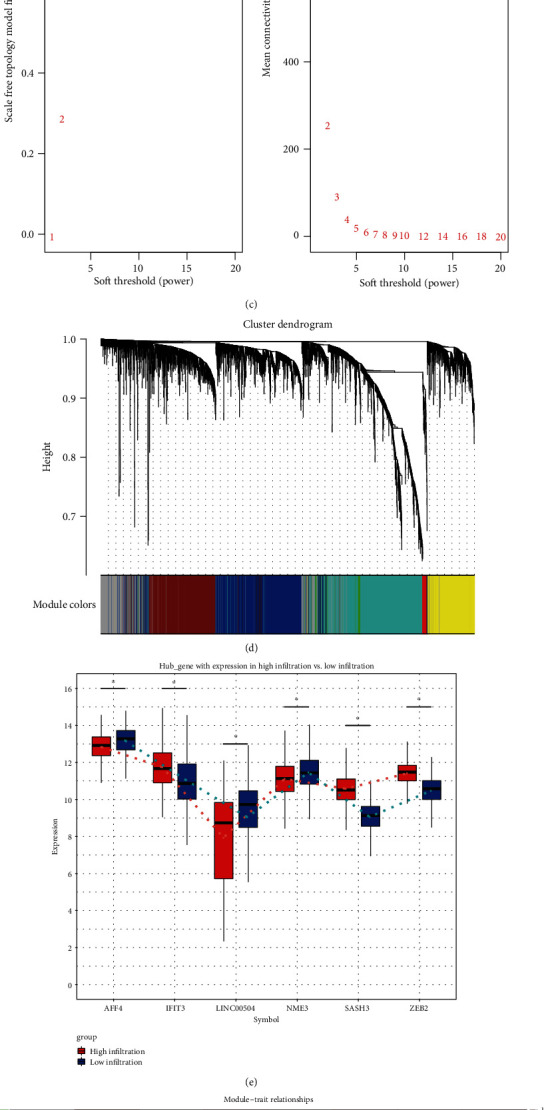
Coexpression of differentially expressed genes between high and low immune cell infiltration. (a) Differentially expressed genes between high and low immune cell infiltration in TCGA, GSE37751, GSE42568, and GSE7390. (b) Screening the genes in TCGA with the same up or down direction in other datasets. (c) Determination of soft threshold. (d) The differentially expressed genes were clustered into 6 coexpression modules. (e) The hub gene for each module. (f) The correlation between modules and immune cells. (g) ROC curve of hub genes. (h) The correlation between the SASH3 of module 1 and T cells. ^∗^*P* < 0.05.

To identify the DEGs in breast cancer between high and low immune groups, we screened four groups of differentially expressed genes (DEGs) (Figure 2(a)). The coexpression analysis was carried out by obtaining the intersection gene with the same expression direction of DEGs in TCGA (Figure 2(b)). The soft power threshold *β* = 5 was determined by the “SFT $power estimate” function (Figure 2(c)). We detected six modules and the hub gene of each module (Figures 2(d) and 2(e), Table 1). In addition, the correlation between each module and immune cell was calculated (Figure 2(f)). MEturquoise (module 1) had the strongest positive correlation with cytotoxic cells, T cells, B cells, DC, high immune infiltration, and negative correlation with Eosinophils. MEBlue (module 5) had the strongest positive correlation with Eosinophils and had the strongest negative correlation with cytotoxic cells, T cells, B cells, DC, and high immune infiltration. It was worth noting that the AUC value of hub gene SASH3 in module 1 was the highest, which may affect the immune cell infiltration of breast cancer (Figure 2(g)). In addition, the correlation between SASH3 and T cell in breast cancer with high immune infiltration was also higher than that in breast cancer with low immune infiltration (Figure 2(h)).

### 3.3. Go Function and KEGG Pathway of Module Genes

**Figure 3:**
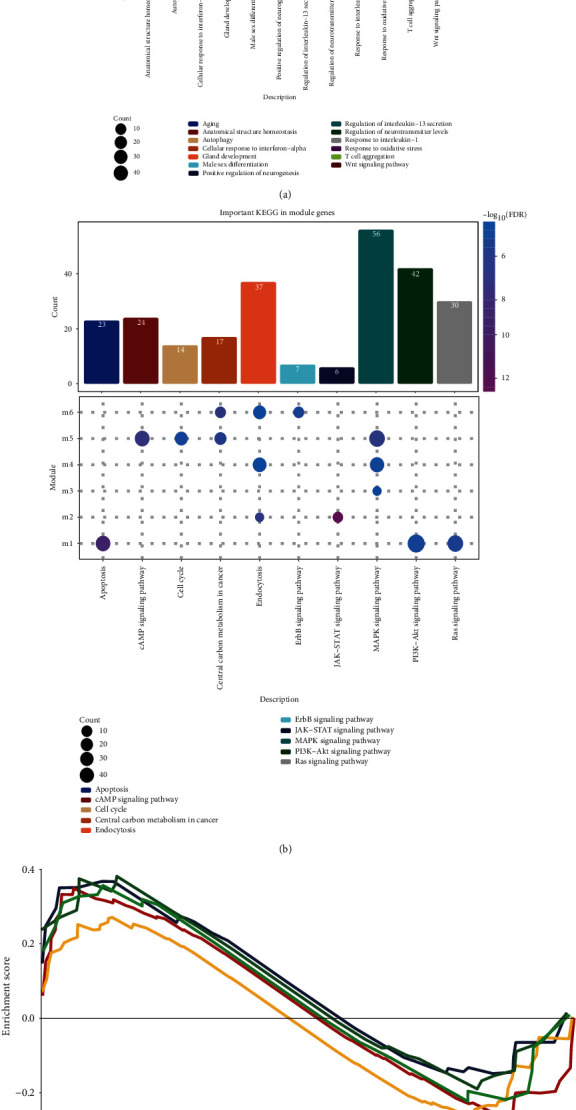
The function and function of module genes. (a) Module genes are involved in biological processes. (b) The KEGG pathway of module genes. (c) The same GSEA KEGG pathway in TCGA, GSE42568, and GSE7390.

Next, in the result of enrichment analysis for module genes, we obtained 3998 biological processes (BP), 411 cell components (CC), and 721 molecular functions (MF). High immune infiltration-related module genes were mainly related to oxidative stress, T cell aggregation, and low immune infiltration-related module genes were mainly related to Wnt signaling pathway (Figure 3(a)). Interestingly, there were a large number of the same terms in the enrichment results of module 1 and module 5, including interleukin-13 secretion, anatomical structure homeostasis, and regulation of neurotransmitter levels. In addition, the module genes enriched 159 KEGG pathways. High immune infiltration-related module genes are mainly related to PI3K Akt signaling pathway, Ras signaling pathway, apoptosis, and low immune infiltration-related module genes are mainly related to cell cycle, MAPK signaling pathway, and cAMP signaling pathway (Figure 3(b)). GSEA results showed that the KEGG pathways related to butanoate metabolism, vitamin digestion, and absorption (Figure 3(c)). These pathways were also verified by GSE42568 and GSE7390.

### 3.4. Mutation Characteristics and Immunophenotype Classification

**Figure 4:**
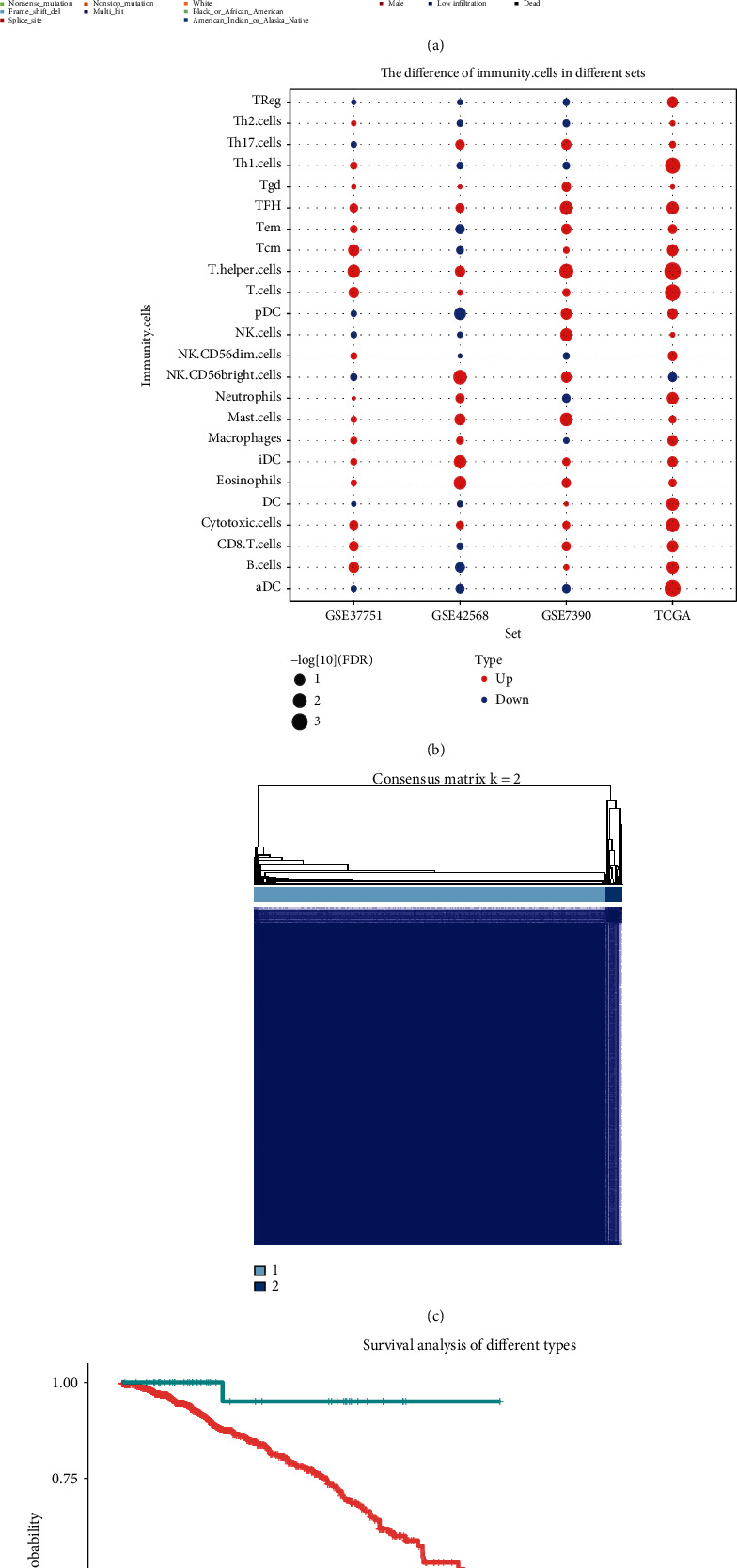
Immunophenotype and mutation characteristics based on 7 key immune genes. (a) Somatic mutations in breast cancer samples with high immune infiltration. (b) The difference of 7 key immune cells in the survival time for more than 5 years and less than 5 years. (c) Breast cancer was classified into two groups according to seven immune cells. (d) Survival difference between the two types of breast cancer.

Furthermore, we observed the distribution of somatic mutations in samples with high or low immune cell infiltration. We found the top 20 mutations related to immune environment; TP53 was the dominant gene (Figure 4(a)). The difference of cytotoxic cells, Eosinophils, iDC, T cells, and T helper cells were verified by other three datasets in the survival time of more than 5 years and less than 5 years (Figure 4(b)). Then, based on the 7 immune cell types, the TCGA queues were classified into two groups (Figure 4(c)). There was a significant difference in OS between the two groups (Figure 4(d)).

## 4. Discussion

In this study, the immune clusters described depend on the abundance of immune infiltration and were independent of other prognostic factors. We also analyzed the coexpression of DEGs in different immune clusters and screened the key genes related to important immune cells. In addition, we provided a new immune-related subtype in breast cancer. In view of the current clinical development of immunomodulatory therapy, this is of interest.

First, we observed the clustering of immune cells in TCGA, GSE37751, GSE42568, and GSE7390. Most of the immune cells were highly expressed in the samples with high immune infiltration. In particular, the results showed that T cells were the protective factor for OS of breast cancer. After identification, the increased of T cell density was related to the improvement of survival rate for breast cancer patients. T cells played an effective role in trying to eliminate tumors [24]. Overall, the infiltration of CD8+ T cells was related to the improvement of clinical prognosis [25]. CD8+ T cell infiltration was associated with good prognosis of ER- and ER+/HER 2+ tumors [8]. The decrease of HER 2 T cell immune level was considered as a prognostic indicator of the increased risk of treatment failure in patients with invasive breast cancer [26].

There were 6 coexpression modules for the DEGs between high and low immune cell infiltration, each module may represent different mechanism of action. Module 1 had the highest positive correlation with T cells and high immune infiltration. The correlation between the T cells and the hub gene SASH3 of module 1 was also higher in the high immune infiltration group than in the low immune infiltration group. It was suggested that SASH3 may affect the immune microenvironment of breast cancer patients. Some studies had shown that SASH3 was a potential prognostic factor for breast cancer patients [27]. As a Lymphocyte specific immune recruitment gene (LYM) gene, SASH3 was related to lymphocytic infiltrating tumor and had good prognosis of breast cancer [28, 29].

In addition, biological function analysis found that module 1 genes were mainly related to oxidative stress, T cell aggregation, and other biological processes. Oxidative stress played an important role in tumor therapy. Cytotoxic therapy increased oxidative damage, which may kill tumor cells [30]. High oxidative stress created a challenging microenvironment for breast cancer metastasis [31]. However, the genes of module 5, which was negatively correlated with high immune infiltration, were mainly involved in Wnt signaling pathway. It had been proved that the atypical activation of Wnt signaling pathway promoted the occurrence and development of tumor, including cell proliferation, migration, invasion, angiogenesis, and resistance to chemotherapy [32]. Wnt pathway activation enhanced the radiation resistance of mouse breast and human breast cancer cell progenitors. It may regulate the number of stem cells and progenitors, making Wnt pathway produced drug resistance in the current anticancer treatment [33]. In the KEGG pathway related to module 1, promoting apoptosis was an important means in the treatment and prevention of breast cancer [34]. The increased MAPK activity associated with module 5 increased the proliferation and migration of breast cancer cells [35]. GSEA results showed that butanoate metabolism was highly enriched in high immune infiltration. The change of butanoate metabolism in breast cancer patients receiving chemotherapy [36]. Changes in metabolic pathways may affect the fate of immune cells to regulate immunity [37]. Our results suggested that the metabolic pathway may regulate the prognosis of breast cancer through immune infiltration.

A large number of predicted immunogen mutations may help to identify patients who may benefit from checkpoint blocking and related immunotherapy [38]. The highest mutation frequency of TP53 was found between high and low immune infiltration, which was also confirmed by other studies [39]. It is tempting that, in breast cancer, TP53 mutations associate with perturbations that increase the likelihood to develop an antitumor immune response. We obtained the phenotypic characteristics of the immune clusters by the consistent clustering. The significant difference in survival between the two phenotype samples indicates that our classification criteria may be further studied. There were some limitations in this study. First of all, this study was based on a public database, so the robustness of immune cell and gene characteristics should be further verified in large prospective clinical trials. Second, experimental studies were needed to further elucidate the biological role of T cells and SASH3 markers.

## 5. Conclusion

In this study, TCGA was used as the main analysis data, and GSE37751, GSE42568, and GSE7390 were used to identify the immune microenvironment and characteristic genes with high or low immune cell infiltration. We found that T cell was a protective factor for the prognosis of breast cancer. Coexpression analysis identified the module genes related to immune cell infiltration. As a positive correlation module with high immune infiltration, SASH3 had a high correlation with T cells and better AUC value. The characteristic genes of high and low immune cell infiltration participate in different biological functions and signal pathways to affect the development of breast cancer. There were significant differences in overall survival between the two phenotypes based on 7 protective immune cell clusters. This is of great significance to further improve our understanding of how to manipulate immune TME.

## Figures and Tables

**Table 1:** Hub genes of modules.

Colour	Hub genes	Module
Blue	LINC00504	m5
Brown	AFF4	m4
Green	ZEB2	m3
Red	IFIT3	m2
Turquoise	SASH3	m1
Yellow	NME3	m6

## Data Availability

We collected the original microarray data of breast cancer tissue from The Cancer Genome Atlas (TCGA) and gene expression omnibus (GSE42568, GSE37751and GSE7390.), as well as the relevant clinicopathological data.
